# Supplementary material for: Motility precedes egress of malaria parasites from oocysts
Source: eLife. 2017 Jan 24;6:e19157. doi: 10.7554/eLife.19157 (PMC5262382; doi:10.7554/eLife.19157)
Supplement: Supplementary file 1. — Multiple sequence alignment with TRP1 homologues from P. berghei, P. chabaudi, P. yoelii 17X, P.vivax, P. knowlesi and P. falciparum 3D7. Highly conserved residues are written in white and highlighted in black, mostly conserved residues are highlighted in dark grey and less conserved residues are highlighted in light grey. The N-terminus (not present in gfp-trp1ΔN) is marked with a green line, the thrombospondin repeat is indicated in blue, the transmembrane domain is marked in orange and the C-terminus (not present in gfp-trp1ΔC) is highlighted in yellow. The red line marks a short sequence of 11 amino acids that is duplicated in the tagged lines gfp-trp1ΔN, gfp-trp1ΔC, gfp-trp1comp and gfp-trp1 before and after the GFP to ensure the structural integrity of the protein. DOI: http://dx.doi.org/10.7554/eLife.19157.034 [file elife-19157-supp1.docx]

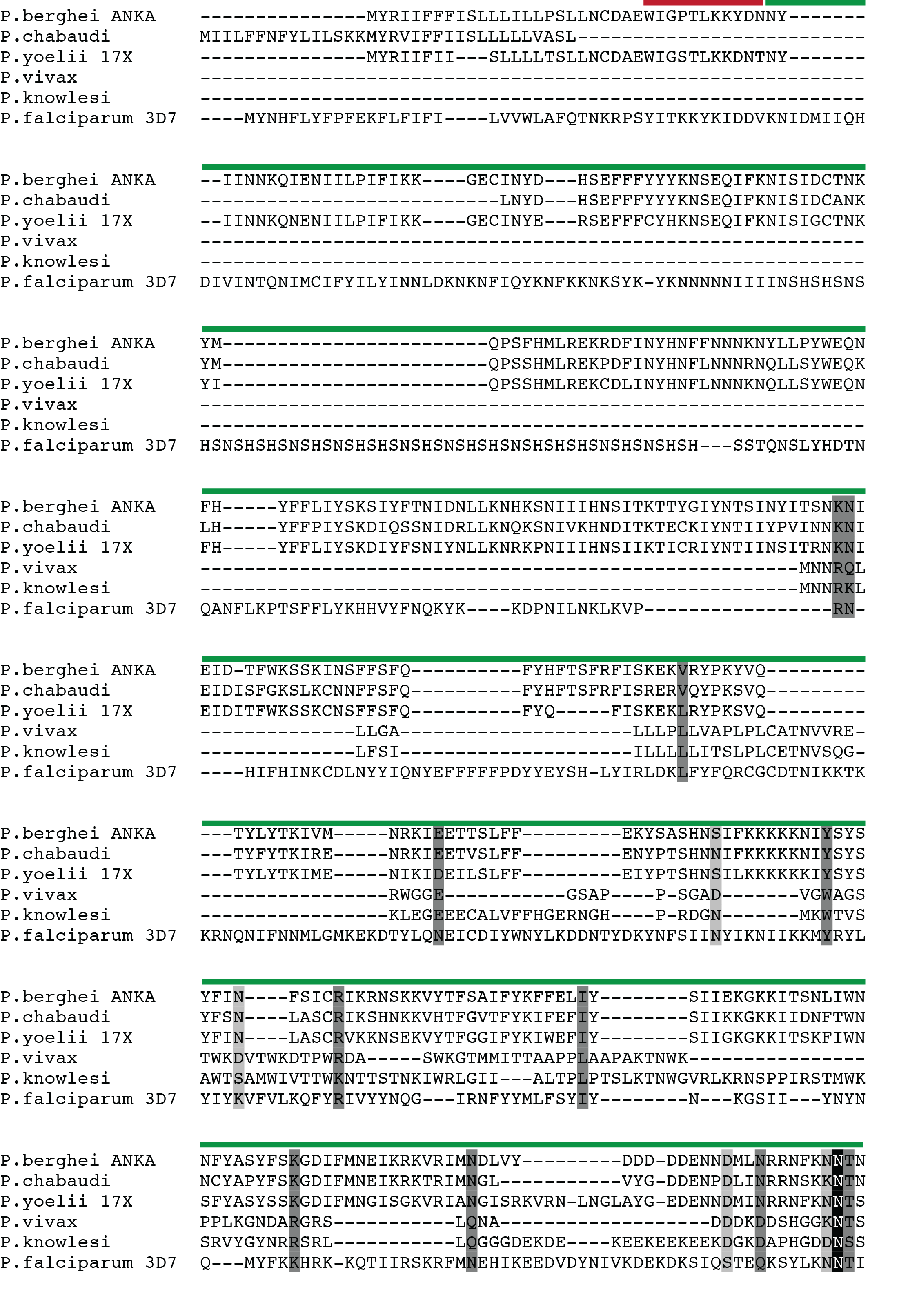


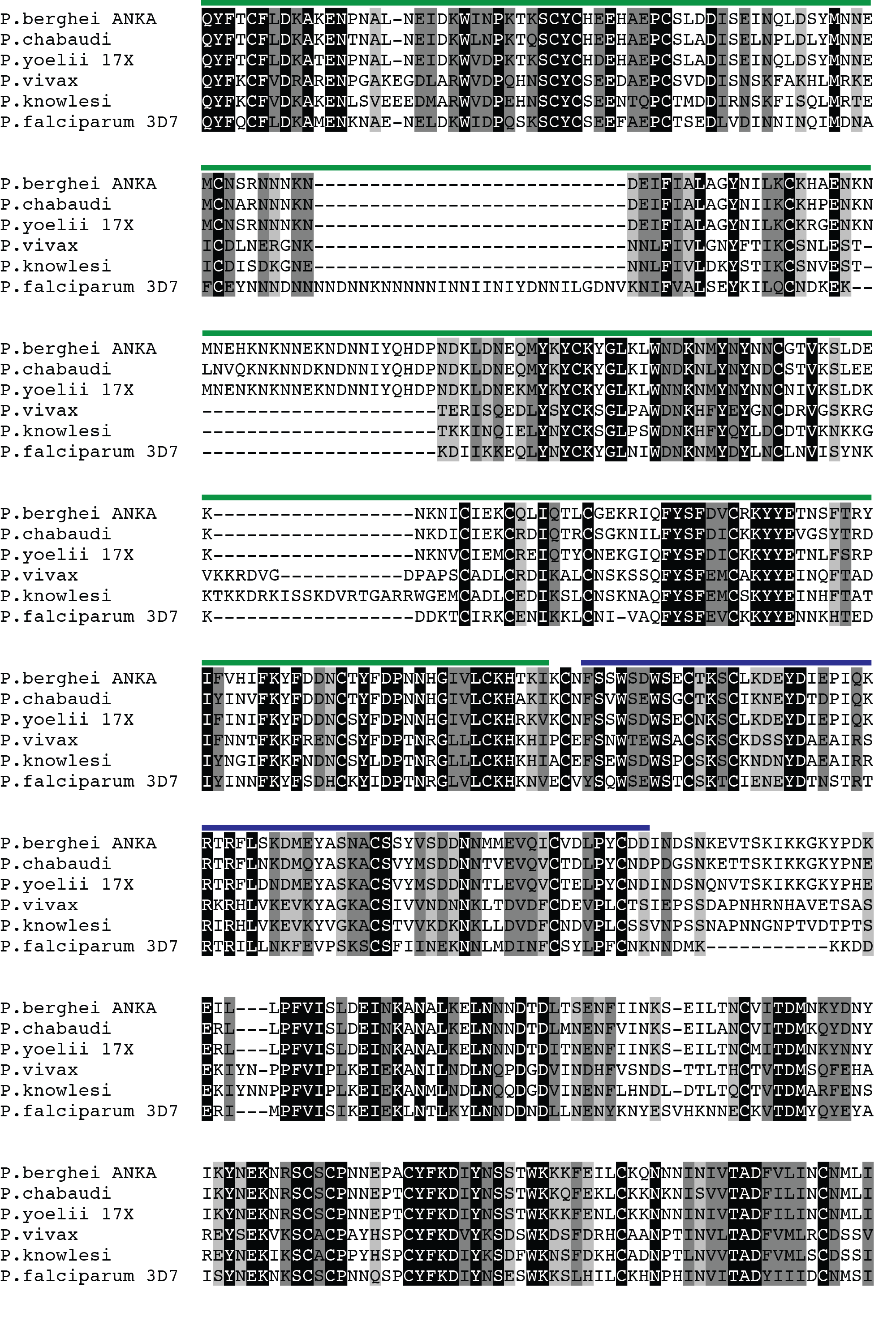


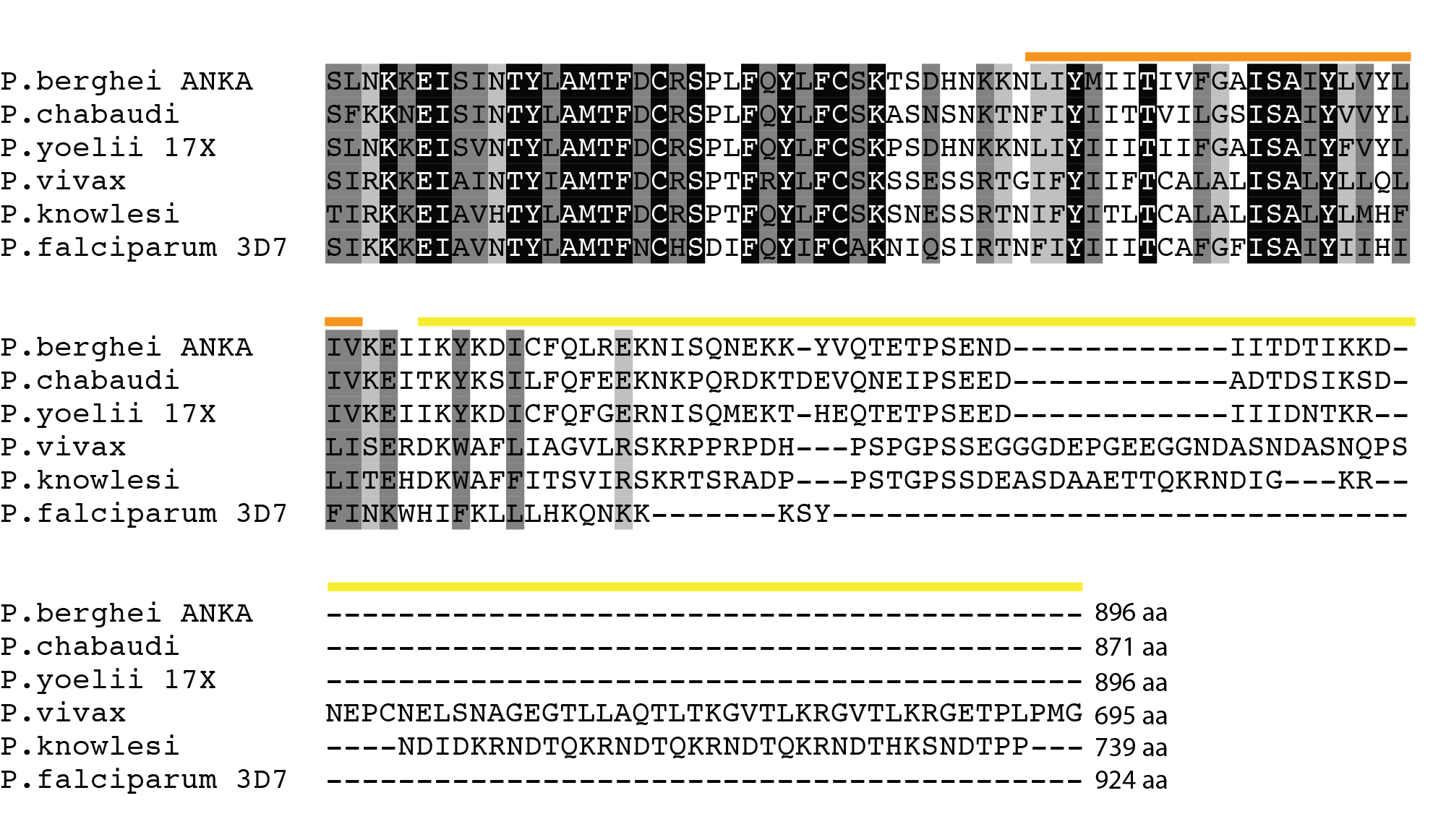


**Supplemental material. Alignment of TRP1 homologues from different *Plasmodium* species**

Multiple sequence alignment with TRP1 homologues from *P. berghei*, *P. chabaudi*, *P. yoelii* *17X*, *P.vivax*, *P. knowlesi*, *P. falciparum 3D7*. Highly conserved residues are written in white and highlighted in black, mostly conserved residues are highlighted in dark grey and less conserved residues are highlighted in light grey. The N-terminus (not present in *gfp-trp1ΔN*) is marked with a green line, the thrombospondin repeat is indicated in blue, the transmembrane domain is marked in orange and the C-terminus (not present in *gfp-trp1ΔC*) is highlighted in yellow. The red line marks a short sequence of 11 amino acids that is duplicated in all tagged lines (*gfp-trp1ΔN, gfp-trp1ΔC, gfp-trp1comp* and *gfp-trp1*) before and after the GFP to ensure the structural integrity of the protein.
